# Prohibitin 2 Regulates the Proliferation and Lineage-Specific Differentiation of Mouse Embryonic Stem Cells in Mitochondria

**DOI:** 10.1371/journal.pone.0081552

**Published:** 2014-04-07

**Authors:** Megumi Kowno, Kanako Watanabe-Susaki, Hisako Ishimine, Shinji Komazaki, Kei Enomoto, Yasuhiro Seki, Ying Ying Wang, Yohei Ishigaki, Naoto Ninomiya, Taka-aki K. Noguchi, Yuko Kokubu, Keigoh Ohnishi, Yoshiro Nakajima, Kaoru Kato, Atsushi Intoh, Hitomi Takada, Norio Yamakawa, Pi-Chao Wang, Makoto Asashima, Akira Kurisaki

**Affiliations:** 1 Graduate School of Life and Environmental Sciences, The University of Tsukuba, Tsukuba, Ibaraki, Japan; 2 Research Center for Stem Cell Engineering, National Institute of Advanced Industrial Science and Technology (*AIST*), Tsukuba, Ibaraki, Japan; 3 Department of Anatomy, Saitama Medical School, Moroyama, Iruma, Saitama, Japan; 4 Department of Biological Science, Graduate School of Science, The University of Tokyo, Meguro, Tokyo, Japan; 5 Department of Life Sciences (Biology), Graduate School of Arts and Sciences, The University of Tokyo, Meguro, Tokyo, Japan; 6 Japan Society for the Promotion of Science (JSPS), Tsukuba, Ibaraki, Japan; 7 Health Research Institute, National Institute of Advanced Industrial Science and Technology (*AIST*), Tsukuba, Ibaraki, Japan; 8 Life Science Center of Tsukuba Advanced Research Alliance, The University of Tsukuba, Tsukuba, Ibaraki, Japan; Kanazawa University, Japan

## Abstract

**Background:**

The pluripotent state of embryonic stem (ES) cells is controlled by a network of specific transcription factors. Recent studies also suggested the significant contribution of mitochondria on the regulation of pluripotent stem cells. However, the molecules involved in these regulations are still unknown.

**Methodology/Principal Findings:**

In this study, we found that prohibitin 2 (PHB2), a pleiotrophic factor mainly localized in mitochondria, is a crucial regulatory factor for the homeostasis and differentiation of ES cells. PHB2 was highly expressed in undifferentiated mouse ES cells, and the expression was decreased during the differentiation of ES cells. Knockdown of PHB2 induced significant apoptosis in pluripotent ES cells, whereas enhanced expression of PHB2 contributed to the proliferation of ES cells. However, enhanced expression of PHB2 strongly inhibited ES cell differentiation into neuronal and endodermal cells. Interestingly, only PHB2 with intact mitochondrial targeting signal showed these specific effects on ES cells. Moreover, overexpression of PHB2 enhanced the processing of a dynamin-like GTPase (OPA1) that regulates mitochondrial fusion and cristae remodeling, which could induce partial dysfunction of mitochondria.

**Conclusions/Significance:**

Our results suggest that PHB2 is a crucial mitochondrial regulator for homeostasis and lineage-specific differentiation of ES cells.

## Introduction

The pluripotent stem cells, such as embryonic stem (ES) cells and induced pluripotent stem (iPS) cells, are regulated by a specific transcription network composed of core transcription factors such as Oct4, Sox2, and Nanog [1], [2]. Recent reports suggested the involvement of other factors, such as mitochondrial functions, in the regulation of stem cells [3], [4]. Previously, we performed proteomic analyses of mouse ES cells and identified prohibitin 2 (PHB2) as one of the highly expressed proteins in pluripotent mouse ES cells [5]. PHB2 is a pleiotropic protein that has been reported to be essential for cell proliferation and development in higher eukaryotes [6], [7], [8]. PHB2 is mainly involved in the functionality of the mitochondrial inner membrane as a protein-lipid scaffold. Some reports also suggested other functions of PHB2, such as transcriptional regulation in the nucleus and cell signaling in the plasma membrane [9].

Recent studies suggested various roles of PHBs in disease pathogenesis. For example, PHBs are involved in cancer growth and metastasis. PHBs are highly expressed in various cancers, such as hepatocellular carcinoma, endometrial hyperplasia, adenocarcinoma, gastric cancer, and breast cancer [10]. PHBs are also involved in inflammatory diseases, such as inflammatory bowel diseases [9]. Therefore, PHBs are considered as important therapeutic targets for clinical applications. In contrast to roles of PHBs in adult tissues, the detailed functions of PHB2 during early development are still unknown. Gene targeting of PHB2 in mice led to embryonic lethality before embryo day 8.5 [8], [11].

In this study, we took advantage of the multiple differentiation abilities of ES cells, and analyzed the roles of PHB2 on the differentiation as well as pluripotency of these cells. We show that PHB2 localized in mitochondria regulates proliferation and lineage-specific differentiation of pluripotent ES cells.

## Results

**Figure 1:**
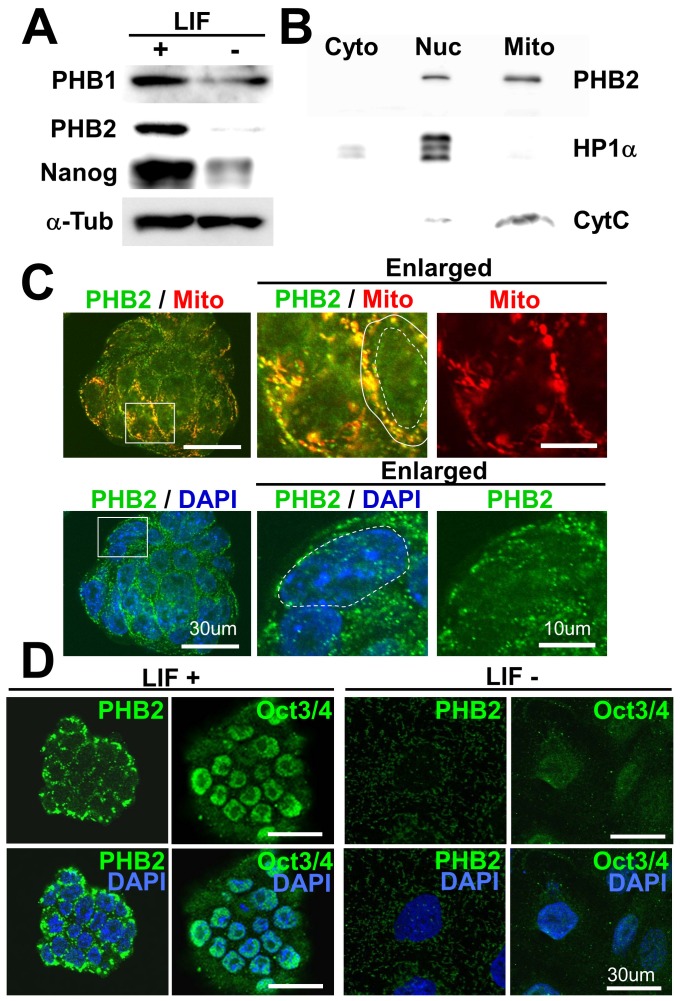
Prohibitin 2 (PHB2) is highly expressed in pluripotent mouse embryonic stem (ES) cells and mainly localized in mitochondria. (**A**) High expression of PHBs in pluripotent ES cells. ES cells cultured with or without LIF for 1 week were subjected to immunoblotting with the indicated antibodies. (**B**) Subcellular fractionation of ES cells cultured in the presence of leukemia inhibitory factor (LIF). The indicated proteins were detected by immunoblotting after separation by SDS-PAGE. (**C**) Localization of PHB2 in pluripotent ES cells. Pluripotent ES cells cultured in the presence of LIF were analyzed by confocal microscopy after immunofluorescence staining with a PHB2 antibody (green). Mitochondria and nuclear DNA were stained with MitoTracker (red) and DAPI (blue), respectively. Enlarged images of the boxed area in the left panels are shown in the middle and right panels. The white solid and dotted circles in the middle panels show the plasma membrane and nuclear envelopes of ES cells, respectively. Scale bar, 10 µm. (**D**) ES cells cultured with or without LIF for 1 week were analyzed by immunofluorescence staining with a PHB2 or Oct4 antibody. Scale bar, 30 µm.

To identify the novel regulatory proteins of ES cells, we have surveyed proteins selectively expressed in pluripotent mouse ES cells by differential proteomic analyses, and identified PHB2 as one of those proteins [5]. Recently, other groups have also reported that PHB proteins are highly expressed in primate ES cells, including in humans [12], [13]. However, the functions of PHBs in ES cells are still unknown. As shown in Fig. 1A, PHB1 and PHB2 are highly expressed in pluripotent mouse ES cells, and their expression was significantly decreased after 1 week of culture without leukemia inhibitory factor (LIF). PHB2 was much more decreased than PHB1. PHB2 is mainly localized in the mitochondria of pluripotent mouse ES cells (Fig. 1C). However, PHB2 signal was also detected in the nucleus. Subcellular fractionation of pluripotent ES cells further confirmed the localization of PHB2 in both fractions (Fig. 1B). When mouse ES cells were cultured in the absence of LIF for 1 week, the cells lost the expression of the pluripotency-specific marker Oct4. They were morphologically differentiated into flattened cells, and the expression of PHB2 was diminished (Fig. 1D).

**Figure 2:**
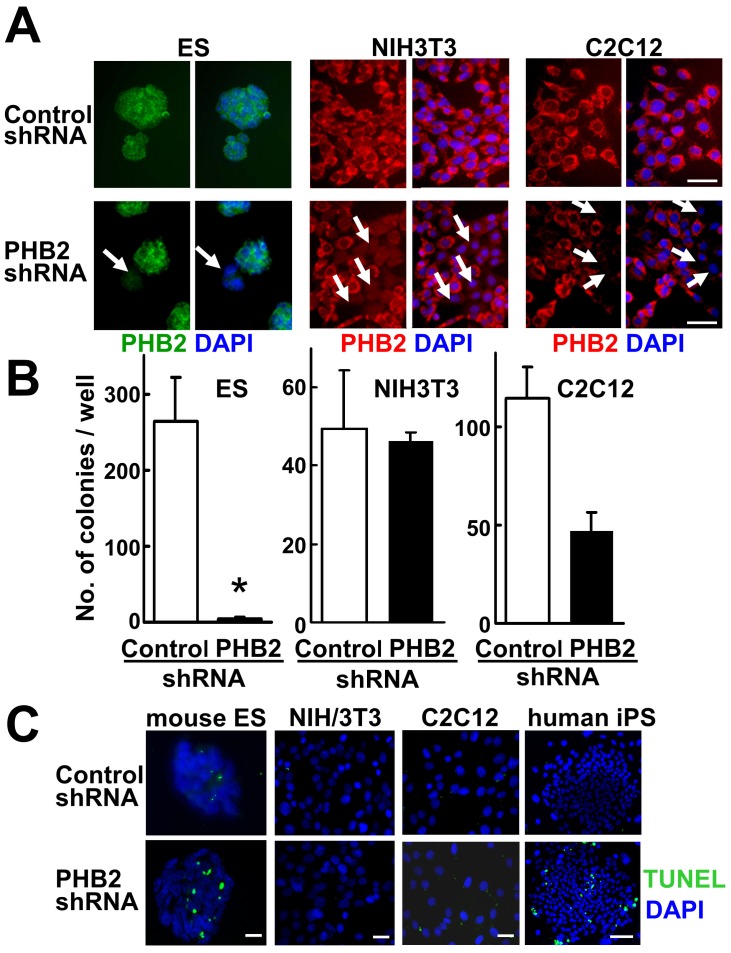
Knockdown of PHB2 in ES cells causes induction of apoptosis. (**A**) Knockdown of endogenous PHB2 in mouse ES, NIH3T3, and C2C12 cells. Cells transiently transfected with a PHB2 shRNA-expressing plasmid were fixed 2 days after transfection and immunostained with a PHB2 antibody. White arrows in the pictures indicate PHB2-knockdown cells. Nuclei were stained with DAPI. Scale bar, 50 µm. (**B**) ES cells stably expressing PHB2 shRNA cannot be established. ES, NIH3T3, and C2C12 cells were infected with a PHB2 shRNA retrovirus, and the stable clones were selected for 10 days in the presence of 500, 300, or 1000 µg/ml G418, respectively. The numbers of the established clones were counted. (**C**) TUNEL staining of PHB2-knockdown cells. Mouse ES, NIH3T3, C2C12, and human iPS cells were transiently transfected with the PHB2 shRNA-expressing vector and subjected to TUNEL staining after 2 days culture. TUNEL, green; DAPI, blue. Scale bar, 100 µm.

To examine the functions of endogenous PHB2 in ES cells, we generated a PHB2-specific shRNA-expressing retrovirus vector. As shown in Fig. 2A (left), transient transfection of the PHB2 shRNA vector successfully knocked down endogenous PHB2 in mouse ES cells. However, ES cell lines stably expressing PHB2 shRNA could not be established. In contrast, the control shRNA-expressing ES cells were readily obtained (Fig. 2B, left), suggesting that *PHB2* is an indispensable gene for the survival of pluripotent ES cells. As shown in Fig. 2C (left), transient transfection of a PHB2 shRNA-expressing vector significantly induced terminal deoxynucleotidyl transferase-mediated deoxyuridine triphosphate nick end-labeling (TUNEL)-positive cells among the mouse ES cells, suggesting that knockdown of PHB2 induced apoptosis in ES cells. In contrast, non-ES cell lines (NIH3T3 and C2C12) that stably express PHB2 shRNA were easily established (Fig. 2B, middle and right). No apparent apoptosis was observed (Fig. 2C, middle), although efficient knockdown of endogenous PHB2 was equally observed in these differentiated cells (Fig. 2A, middle, right). This ES-specific induction of apoptosis by the knockdown of PHB2 was also observed in human pluripotent stem cells (Fig. 2C, right). These results suggest that high-level expression of PHB2 is indispensable for the maintenance of pluripotent ES cells.

**Figure 3:**
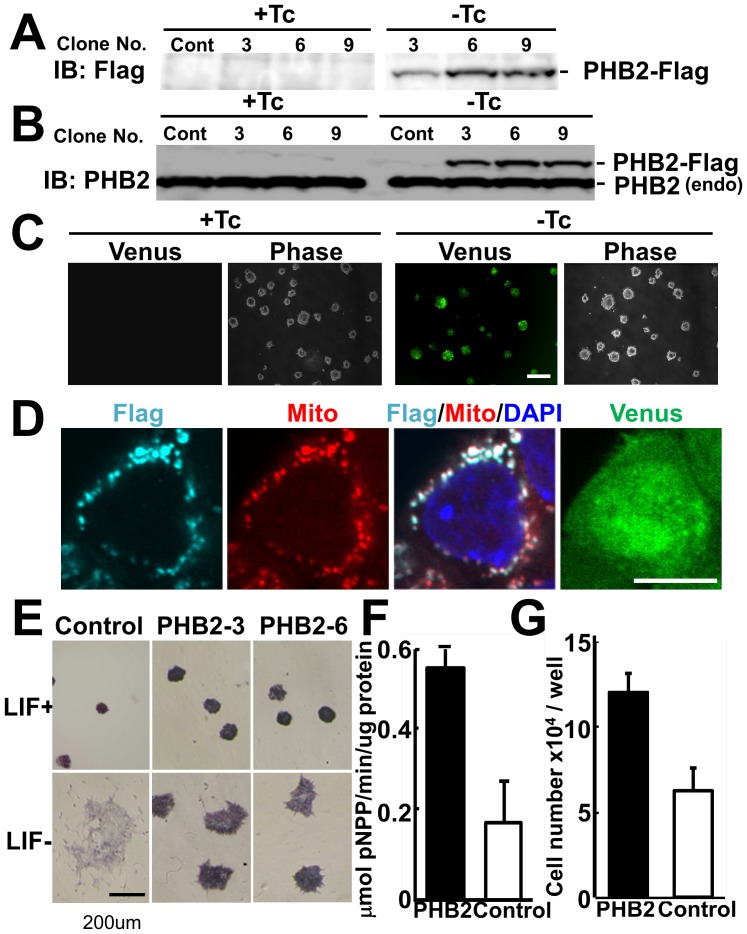
Ectopic expression of PHB2 promotes cell proliferation of mouse pluripotent mouse ES cells. (**A, B**) Ectopic expression of PHB2 under the control of a Tet-off promoter. EBRTcH3 cells carrying the *PHB2* target gene under the control of a tetracycline (Tc)-regulated promoter were cultured with or without Tc for 4 days, and subjected to immunoblotting either with a Flag (A) or a PHB2 antibody (B). (**C**) Expression of Venus under the control of a Tet-off promoter. Venus protein was expressed simultaneously with PHB2 under the same Tet-off promoter. A PHB2-expressing clone was cultured with or without Tc for 4 days. Scale bar, 300 µm. (**D**) Confocal microscopic images of the ectopically expressed PHB2-Flag protein in ES cells. PHB2-expressing clones cultured without Tc for 2 days were immunostained with Flag antibody (cyan). Mitochondria were stained with MitoTracker (red), and nuclei were stained with DAPI (blue). Merged regions of cyan and red are shown as white areas. Autofluorescence of Venus induced upon withdrawal of Tc from the culture medium was also observed. Scale bar, 10 µm. (**E**) Alkaline phosphatase staining of PHB2-expressing ES cells. PHB2-expressing ES cells cultured without LIF for 2 days were cultured for 4 more days in the absence of LIF and Tc. The cells were fixed and analyzed for alkaline phosphatase activity. (**F**) Liquid assay for alkaline phosphatase activity. The ES cells cultured as in (E) were subjected to the liquid assay for alkaline phosphatase. (**G**) Growth rate of PHB2-expressing ES cells. ES cells cultured without LIF and Tc for 2 days were seeded in a 12-well plate at 1×10^4^ cells per well. The cells were cultured for 3 more days without LIF, and the number of cells was counted. The experiments were done in duplicate and repeated twice. P<0.05.

Next, PHB2-expressing ES clones were established by using a Tet-off expression system [14]. These ES clones expressed PHB2-Flag protein when they were cultured in the absence of tetracycline (Tc) (Fig. 3A). Exogenous PHB2-Flag protein was slightly larger than the endogenous PHB2, and was expressed in lesser amount than the endogenous PHB2 (Fig. 3B). Culture without Tc simultaneously induced the expression of a GFP variant protein, Venus, in addition to PHB2, owing to the presence of the *IRES* sequence in the knock-in vector (Fig. 3C). Exogenous PHB2-Flag protein was mostly localized in mitochondria (Fig. 3D). When PHB2-expressing ES cells were cultured in the absence of LIF and allowed to differentiate spontaneously, the cells showed prolonged alkaline phosphatase activity for a while, although the compact morphology of these cells was disrupted. In contrast, ES cells expressing a control vector showed a flattened morphology with significantly decreased alkaline phosphatase activity (Fig. 3E, F). Moreover, the growth of PHB2-expressing ES cells was faster than that of the control vector-expressing cells when cultured in the absence of LIF (Fig. 3G). However, overexpression of PHB2 failed to maintain the pluripotency of ES cells for a longer culture time (data not shown). These results suggest that PHB2 promotes cell proliferation and retards the spontaneous differentiation of pluripotent ES cells.

**Figure 4:**
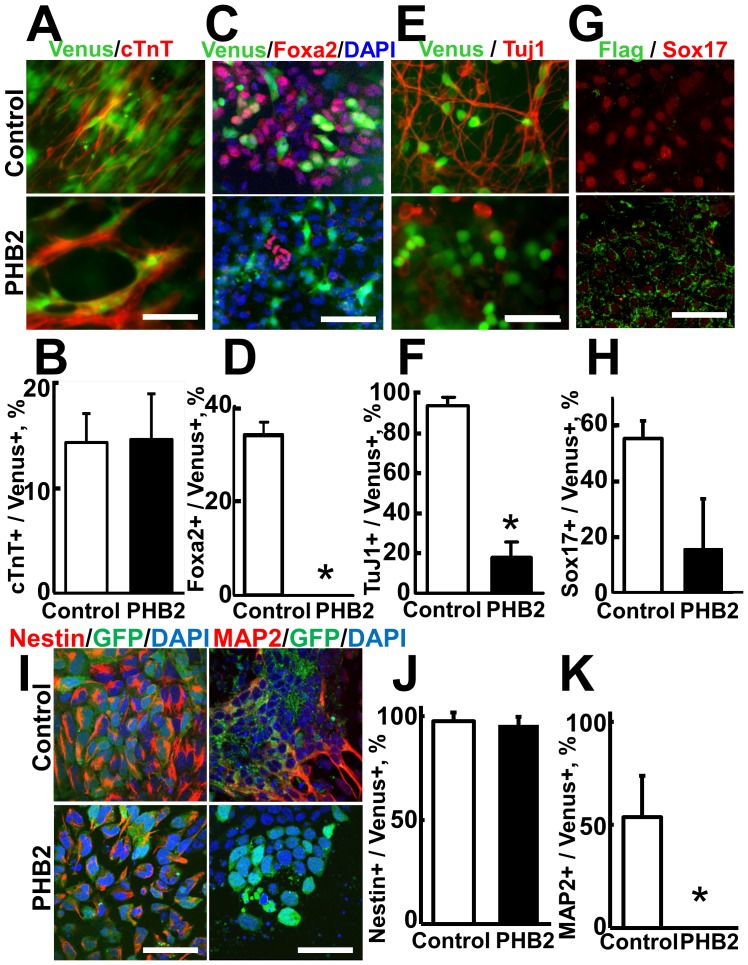
PHB2 inhibits the differentiation of ES cells into neuronal cells and endodermal cells but not into cardiomyocytes. (**A, C, E**) Effect of ectopic expression of PHB2 on the differentiation of mouse ES cells into cardiomyocytes (A), endoderm cells (C), or neuronal cells (E). EBRTcH3 cells were differentiated in the absence of Tc. The differentiated cells were immunostained with the indicated antibodies. The ectopic expression of PHB2 was monitored with the autofluorescence of the coexpressed Venus protein. (**B, D, F**) Quantification of the effect of PHB2 on the differentiation of ES cells. The number of Venus-positive PHB2-expressing cells and indicated marker-positive cells were counted. The bar graph shows the average numbers of immunofluorescently stained cells counted in 3 independent eye fields. 0: No Foxa2-positive cell was detected in Venus-positive cells. Scale bar, 50 µm. (**G**) Inhibitory effect of PHB2 on the differentiation of human iPS cells into Foxa2-positive endodermal cells. The human iPS cell line 201B7 stably expressing PHB2-Flag (Fig. S1A clone 9) was differentiated into the endodermal lineage. The differentiated cells were detected by immunofluorescence staining with Sox17 antibody. Scale bar, 50 µm. (**H**) Quantification of the inhibitory effect of PHB2 on the endodermal differentiation of human iPS cells. The number of Flag-positive PHB2-expressing cells and nuclear-localized Sox17-immunopositive cells were counted. Scale bar, 50 µm. P<0.01. (**I**) PHB2 does not inhibit the differentiation of ES cells into neural stem/progenitor cells. The EBRTcH3 cell line was differentiated as in (A) and immunostained with Nestin or MAP2 antibody. (**J, K**) Quantification of the inhibitory effect of PHB2 in (I).

Next, we examined the effect of ectopic expression of PHB2 on the differentiation of ES cells by using the Tet-off-regulated PHB2-expressing ES cells. First, we tested the effect of PHB2 on the differentiation of ES cells into the mesodermal lineage, particularly into cardiomyocytes. On the 14th day after differentiation, the PHB2-expressing Venus-positive cells efficiently differentiated into cTnT-positive cardiomyocytes similar to the control cells (Fig. 4A, B), suggesting that PHB2 does not affect the differentiation of ES cells into the cardiomyocyte lineage.

In contrast, when the ES cells were differentiated into endodermal cells with a high concentration of activin, significant induction of Foxa2-positive endodermal cells was observed. However, PHB2-expressing Venus-positive cells did not differentiate into endodermal cells that show the characteristic nuclear localization of Foxa2 (Fig. 4C, D). A similar inhibitory effect was also observed in human pluripotent stem cells (Fig. 4G, H).

When the ES cells were induced to differentiate into neuronal lineages by using the SFEB method [15], the Venus-positive control ES cells efficiently differentiated into TuJ1-positive neuronal cells. In contrast, most of the PHB2-expressing Venus-positive cells showed significantly fewer TuJ1-positive cells (Fig. 4E, F). Interestingly, PHB2-expressing cells expressed the neural stem cell marker nestin, but not the dendrite marker MAP2 after the neuronal differentiation of ES cells (Fig. 4I–K), suggesting that PHB2 particularly inhibits differentiation of ES cells after neural stem/progenitor cells. These results indicate that ectopic expression of PHB2 strongly inhibits the differentiation into the ectodermal and endodermal cell lineages of pluripotent stem cells.

**Figure 5:**
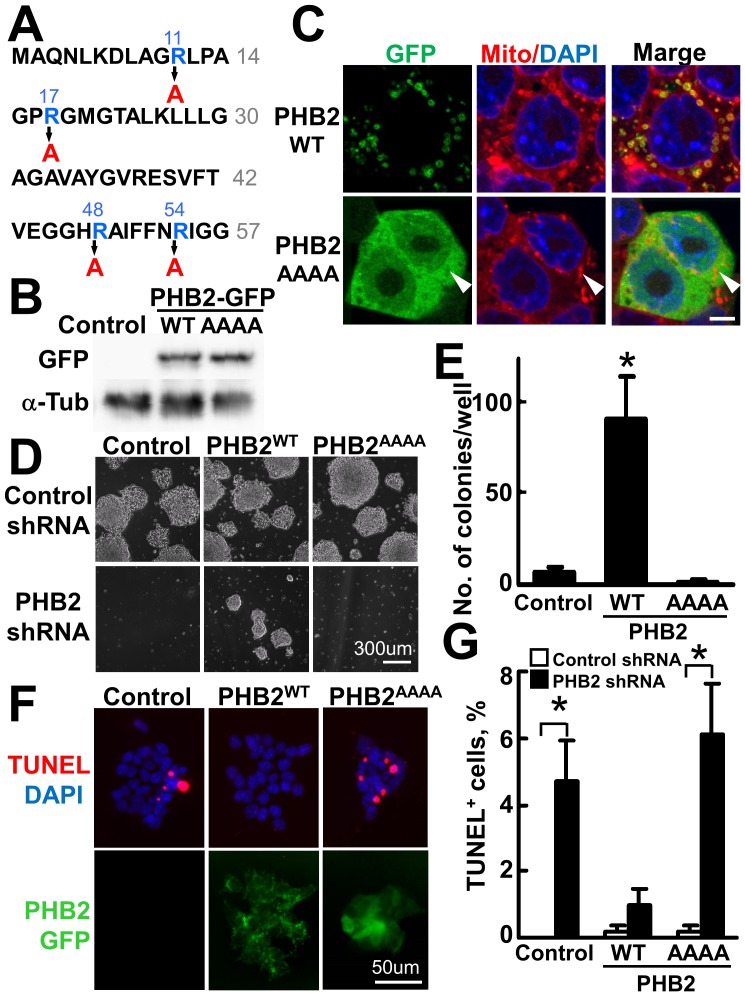
PHB2 localized in mitochondria is essential for the survival of pluripotent ES cells. (**A**) N-terminal sequence of the mitochondria-targeting signal mutated version of PHB2. (**B**) Establishment of shRNA-insensitive PHB2-GFP stable clones in mouse ES cells. The wild type and the mitochondria-targeted signal-mutated version of PHB2-expressing ES clones were established. The expression of these proteins was monitored by immunoblotting with a GFP antibody. (**C**) Subcellular localization of PHB2-GFP^WT^ and PHB2^AAAA^-GFP proteins in pluripotent ES cells. Confocal images of GFP-tagged PHB2 in ES cells are shown. Arrowheads indicate the location of mitochondria in ES cells. PHB2^AAAA^-GFP but not PHB2-GFP^WT^ failed to localize in mitochondria. Scale bar, 5 µm. (**D, E**) PHB2^WT^ but not PHB2^AAAA^ rescued cells from apoptosis induced by PHB2 knockdown in ES cells. ES cells expressing the PHB2^WT^ or PHB2^AAAA^ transgene (1×10^4^ cells) were infected with PHB2 shRNA- or the control shRNA-expressing retrovirus, and cultured in the presence of 500 µg/ml G418 for 10 days (D). The resulting numbers of colonies counted in 5 different eye fields are shown in (E). (**F, G**) TUNEL staining of PHB2-knockdown ES cells rescued by the exogenous mitochondrial PHB2 gene. ES cells expressing the control vector or shRNA-insensitive PHB2-GFP (1×10^4^ cells) were transfected with PHB2 shRNA vector and cultured for 3 days. The apoptotic cells were detected by TUNEL staining (F), and the numbers of cells were counted (G). Knockdown of endogenous PHB2 induced TUNEL-positive apoptosis. Cells were rescued from apoptosis by ectopic expression of PHB2^WT^ but not PHB2^AAAA^.

To investigate the mechanism of these observations, we generated stable ES clones that express shRNA-insensitive wild-type PHB2 or the mitochondria-targeted signal-mutated version of PHB2 (PHB2^AAAA^) [8] as the GFP fusion protein (Fig. 5A, B). PHB2^WT^-GFP was specifically localized in mitochondria (Fig. 5C). In contrast, PHB2^AAAA^-GFP was mainly localized in the cytoplasm, and a lesser amount of this mutant was detected in the nucleus. PHB2^AAAA^-GFP failed to be transported into mitochondria (Fig. 5C, arrowhead). When ES cells were infected with the PHB2 shRNA retrovirus, none of the cells survived after G418 selection (Fig. 5D, left). The ES cells started to undergo apoptosis 2 days after the induction of PHB2 shRNA. Cell numbers were gradually decreased by apoptosis, and dead cells gradually detached from the culture plate. Most cells die in approximately 5–6 days. The cell death induced by the knockdown of endogenous *PHB2* gene was reversed by the ectopic expression of the shRNA-insensitive *PHB2^WT^-GFP* gene. In contrast, the shRNA-insensitive *PHB2^AAAA^-GFP* gene failed to inhibit the knockdown-induced cell death (Fig. 5D, E). TUNEL staining of these cells further confirmed that effective suppression of apoptosis was attained by the ectopic expression of PHB2^WT^-GFP but not the PHB2^AAAA^-GFP mutant (Fig. 5F, G). These results demonstrated that PHB2 localized in mitochondria is essential for the survival of pluripotent ES cells.

**Figure 6:**
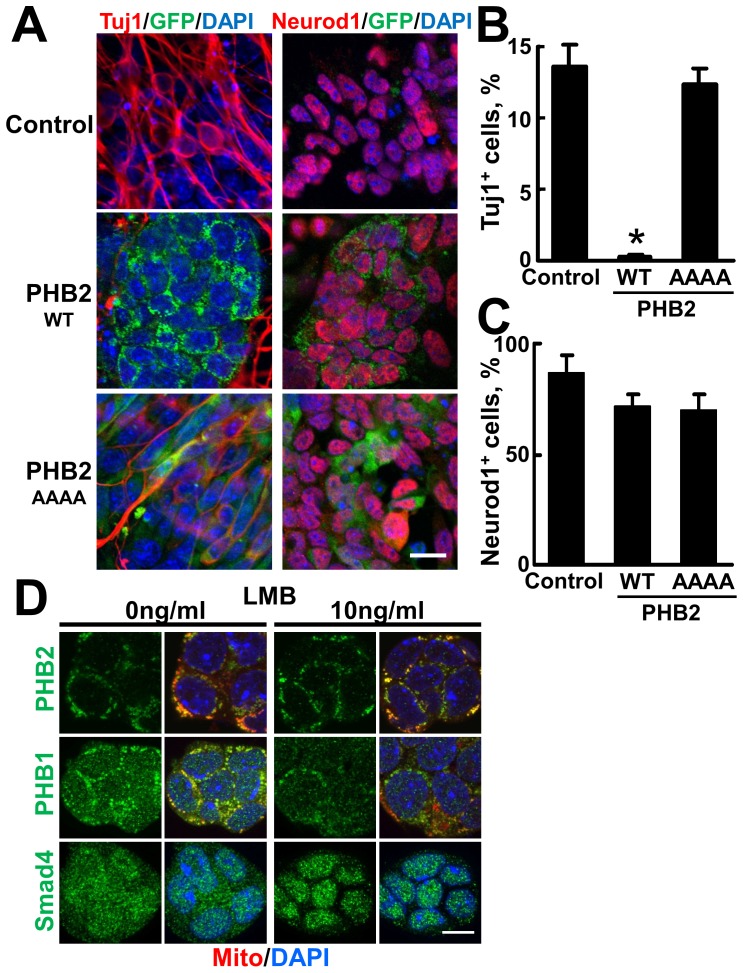
PHB2 localized in mitochondria inhibits neuronal differentiation of ES cells. (**A–C**) Ectopic expression of PHB2^WT^ but not PHB2^AAAA^ inhibits neuronal differentiation. ES cells expressing PHB2-GFP were differentiated into neuronal cells as described in Fig. 4E. (A) The differentiated cells were immunostained with either a Tuj1 or a Neurod1 antibody. Scale bar, 20 µm. (B) Tuj1-positive or (C) Neurod1-positive cells were counted in more than 5 different eye fields. (**D**) Endogenous PHBs do not shuttle in ES cells. Mouse ES cells were treated with a nuclear export inhibitor, LMB, at 10 ng/ml for 8 h. The nuclear-cytoplasm shuttling protein Smad4 was used as a positive control for LMB. Scale bar, 10 µm. Similar results were obtained by LMB treatment at 10 ng/ml for 1 h (data not shown).

The importance of PHB2 in mitochondria was further examined during the differentiation of ES cells. When ES cells expressing the PHB2-GFP genes were differentiated into TuJ1-positive neuronal cells, a strong inhibition of neuronal differentiation was observed in PHB2^WT^-GFP-expressing cells. In contrast, ectopic expression of PHB2^AAAA^-GFP did not inhibit neuronal differentiation at all (Fig. 6A, left, and 6B). Interestingly, PHB2^WT^ did not show an obvious inhibitory effect on the differentiation of ES cells into Neurod1-positive neural stem/progenitor cells (Fig. 6A, right, and 6C). These results suggest that PHB2 localized in mitochondria negatively regulates the differentiation into neurons after the differentiation of ES cells into neural stem/progenitor cells.

A recent study reported that the subcellular localization of PHB1 is regulated by a nuclear exporter, CRM1, with its nuclear export signal located in its C-terminal [16]. Treatment of cells with LMB, an inhibitor of CRM1, abrogated the nuclear export of PHB1, and endogenous PHB1 was concentrated in the nucleus. In mouse ES cells, however, neither PHB1 nor PHB2 showed nuclear localization after treatment with LMB. LMB was effective on ES cells because a transcription factor, Smad4, which shuttles between the nucleus and the cytoplasm [17], was trapped in the nucleus (Fig. 6D). Therefore, our results suggest that PHB2 constitutively localized in mitochondria functions as a crucial regulator for proliferation and cell type–specific differentiation of ES cells.

**Figure 7:**
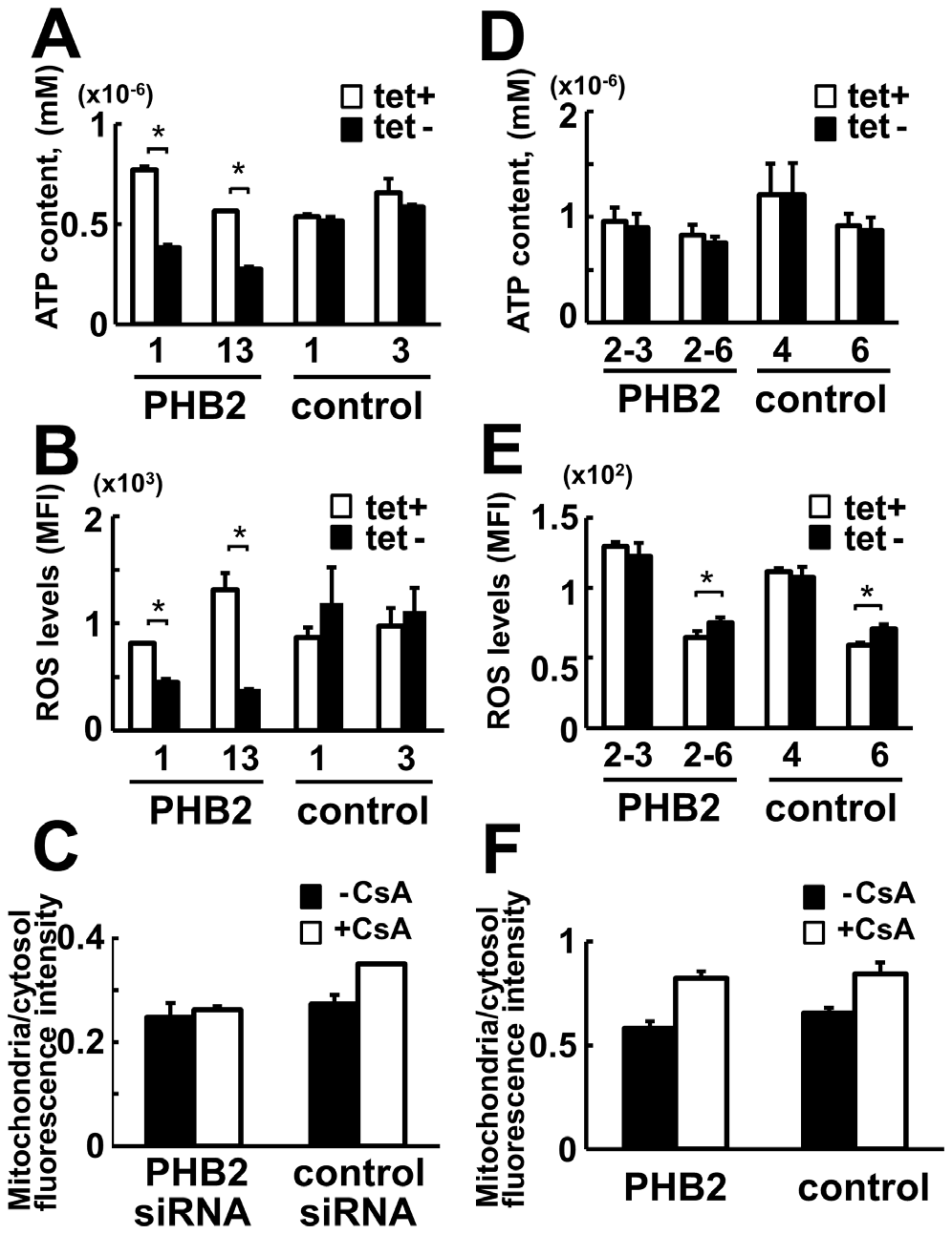
ATP biosynthesis, ROS production, and mitochondrial membrane potential of PHB2-knockdown ES cells and PHB2-overexpressing ES cells. (**A, B**) ATP concentration (A) and ROS level (B) were measured with EBRTcH3 cells that express PHB2 shRNA under the control of a Tc promoter. The ES cells were cultured in either the absence or presence of Tet for 4 days. (**C**) Mitochondrial membrane potential in ES cells transiently transfected with PHB2 siRNA or control siRNA. The ES cells were cultured for 45 h, and treated with or without cyclosporin A (CsA). (**D–F**) ATP concentration (D), ROS level (E), and mitochondrial membrane potential (F) were measured in EBRTcH3 cells that express PHB2 under the control of Tc promoter in the presence of LIF. The ES cells were cultured either absence or presence of Tc for 3 days.

When we analyzed the intracellular ATP level (Fig. 7A), it was apparently decreased upon knockdown of PHB2. The reactive oxygen species (ROS) level was also significantly decreased in PHB2-knockdown cells (Fig. 7B). A membrane-permeable indicator for mitochondrial membrane potential, 5,5′,6,6′-tetrachloro-1,1′,3,3′-tetraethylbenzimi-dazolylcarbocyanine iodide (JC-1), revealed a decrease of membrane potential in PHB2 knockdown cells (Fig. 7C). However, ES cells overexpressing PHB2 did not show a clear difference in ATP, ROS, and mitochondrial membrane potential when they were cultured in the presence of LIF (Fig. 7D–F). These results suggest that ectopic expression of PHB2 may not drastically affect mitochondrial biogenesis in pluripotent ES cells.

Mitochondria in ES cells are small and morphologically immature. Time-lapse confocal microscopy revealed that overexpression of PHB2 in ES cells did not change the size of mitochondria nor did it induce any significant network formation as observed in differentiated cells (Movie S1, S2) [18]. Both PHB2-overexpressing and control cells showed active mitochondrial fusion and fission, indicating that overexpression of PHB2 did not significantly affect mitochondrial dynamics. In contrast, PHB2 knockdown caused mitochondrial swelling (Movie S3, S4) and significantly impaired mitochondrial movement. These results indicate that knockdown of endogenous PHB2 significantly deteriorates mitochondrial dynamics.

**Figure 8:**
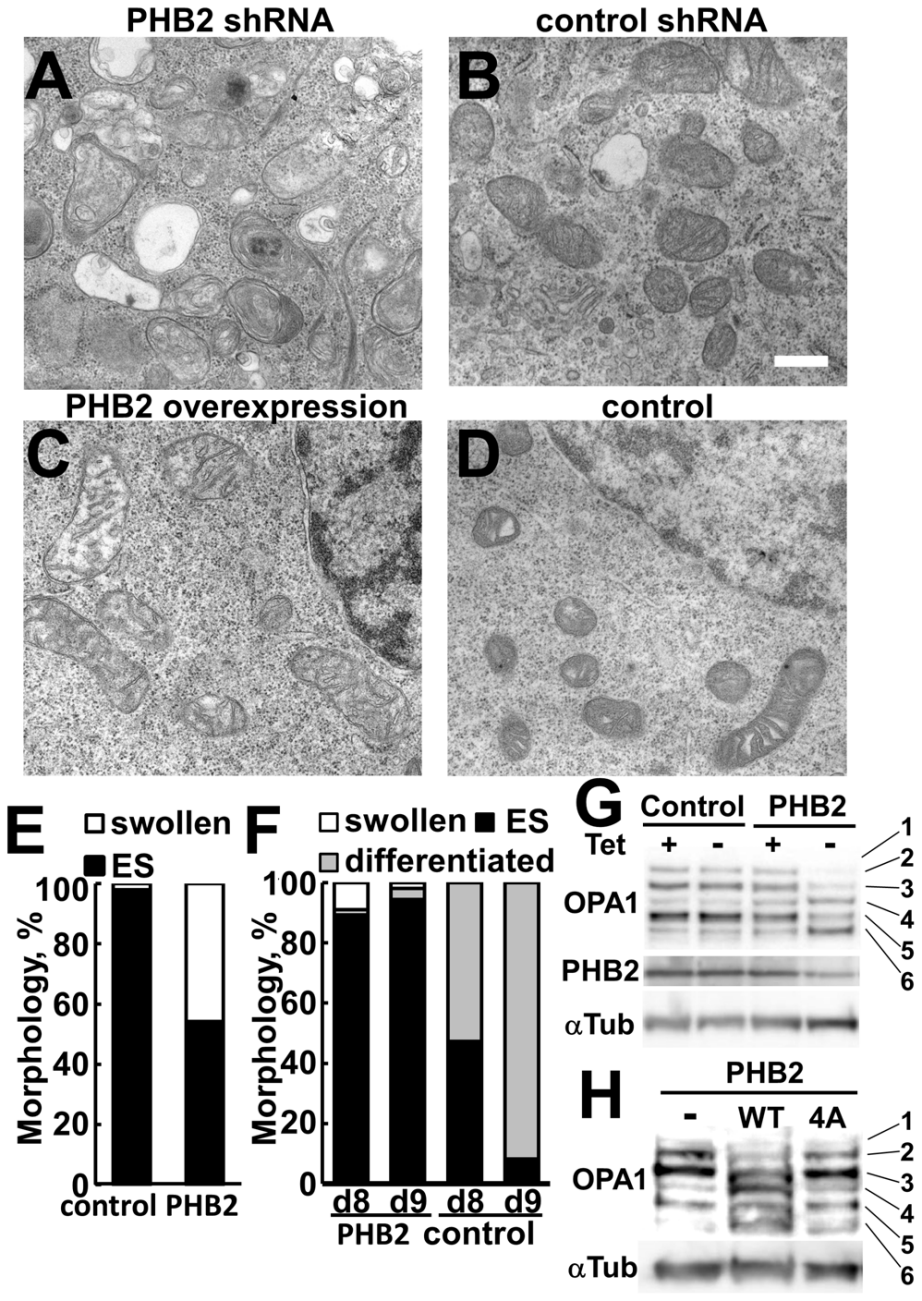
Ultrastructural analysis of mitochondria in PHB2-overexpressing ES cells and PHB2-knockdown ES cells by electron microscopy. (**A**) Mouse ES cells (EBRTcH3) expressing with PHB2 shRNA. (**B**) ES cells (EBRTcH3) expressing a control shRNA. (**C**) Mouse D3 cells overexpressing PHB2. (**D**) D3 cells transfected with a control vector. Scale bar, 500 nm. (**E**) Semiquantitation of morphological differences of mitochondria in D3 cells stably expressing PHB2 or control vector. More than 100 mitochondria were analyzed in these cells. (**F**) Semiquantitation of morphological differences of mitochondria after neuronal differentiation of D3 cells stably expressing PHB2. The ES cells used in (E) were differentiated into the neuronal lineage by using the SFEB method for 9 days, and the samples on day 8 (d8) and day 9 (d9) were subjected to electron microscopy. More than 100 mitochondria were analyzed in these cells. (**G, H**) Immunoblotting of OPA1 in ES cells. Whole lysate of EBRTcH3 cells that express PHB2 shRNA (G) or D3 cells that express PHB2-Flag (H) were analyzed by immunoblotting with OPA1 antibody. α-Tubulin was used as a loading control.

To further investigate the mechanisms of PHB2 on the regulation of ES cells, we analyzed the ultrastructure of mitochondria to determine the effects of PHB2 knockdown and overexpression in mouse ES cells. Knockdown of PHB2 by PHB2-specific shRNA caused significant changes in the morphology of mitochondria, which degenerated into vacuole-like structures and lost cristae (Fig. 8A). Moreover, many lysosomes were observed in the cytoplasm, suggesting that defective mitochondria are degraded by autophagosomes. These phenomena were not observed in control shRNA-expressing cells (Fig. 8B). On the other hand, overexpression of PHB2 induced mitochondrial swelling (Fig. 8C–E). However, these mitochondria still maintained a basic structure, including cristae, which suggests that the mitochondria in PHB2-overexpressing cells would still be functional in pluripotent ES cells as shown in Fig. 7D–F.

When the ES cells were subjected to neuronal differentiation, most of the mitochondria in control ES cells changed their shape into the elongated and matured form that is characteristically observed in neuronally differentiated cells. In contrast, the mitochondria in PHB2-overexpressing ES cells were mainly of the ES-cell-type immature form (Fig. 8F). These observations suggest that overexpression of PHB2 caused failure in the transformation of the ES cell–specific juvenile form of mitochondria into the differentiated cell-specific mature form.

The dynamin-like GTPase OPA1 has been identified as one of the major PHB2 targets in mitochondria [19]–[21]. OPA1 proteins that exist as multiple isoforms with different sizes in the inner mitochondrial membrane are required for the maintenance of the normal structure of cristae. Deletion of the *PHB2* gene in mouse embryonic fibroblast cells promoted processing of OPA1 and led to selective loss of the long OPA1 isoforms [8]. In this study, knockdown of PHB2 in mouse ES cells also induced loss of the longer OPA isoforms (Fig. 8G), severe dysfunction of mitochondria, and apoptosis. Interestingly, significant decrease of the longer OPA1 isoforms and concomitant increase of the shorter isoforms were also observed in PHB2-overexpressing cells, although the mitochondria maintained functional integrity in plurotent ES cells, as shown in Fig. 7D–F. The accelerated processing of OPA1 in PHB2-overexpressing ES cells suggests potential destabilization of cristae structure, which was observed as mild swelling of mitochondria in PHB2-overexpressing ES cells. This might affect efficient ES cell differentiation. As expected, after neuronal differentiation, only immature mitochondria were detected in PHB2-overexpressing ES cells, while an increasing number of mature mitochondria typical for differentiated cells were observed in the control ES cells (Fig. 8F). These results suggest that overexpression of PHB2 would promote the processing of OPA1 and hamper efficient differentiation of ES cells owing to the partial dysfunction of mitochondria.

## Discussion

In this study, we have shown that PHB2 localized in mitochondria is essential for the survival and proliferation of pluripotent ES cells. Enhanced expression of PHB2 itself promotes proliferation of ES cells. In contrast, decreased expression of PHB2 causes apoptosis in ES cells at a much higher rate than that in differentiated cells. Although we observed prolonged alkaline phosphatase activity in PHB2-expressing ES cells cultured in the absence of LIF, overexpression of PHB2 was not able to maintain the pluripotent state of ES cells in a long-term culture without LIF. Therefore, our results suggest that high-level expression of PHB2 enhances the proliferation of pluripotent ES cells and thereby retards their spontaneous differentiation in the absence of LIF rather than actively maintaining the pluripotent state of these cells.

We have shown that PHB2 also regulates the lineage-specific differentiation of pluripotent ES cells. Enhanced expression of PHB2 strongly inhibited the differentiation of ES cells, especially into neuronal and endodermal cells. Initially, we speculated that PHB2 localized in the nucleus regulates the differentiation of ES cells because PHBs have been reported to modulate gene expression by interacting with several cellular signaling molecules [9], such as estrogen receptor [11], [22], [23] and Akt [24], [25]. Previous studies with C2C12 cells have shown that PHB2 can translocate into the nucleus and inhibit skeletal muscle differentiation by direct interaction with the nuclear transcription factors MyoD and MEF2, and by the recruitment of HDAC1 [24], [25]. Several papers describe the transcriptional repressor function of PHBs in the nucleus [22], [23], [26], [27]. In breast cancer cell lines but not in normal cells, PHB1 is mainly localized in the nucleus and can be exported in response to a topoisomerase 1 inhibitor, camptothecin, in a CRM-1-dependent manner [16]. PHB2 also translocates into the nucleus in response to estrogen, when estrogen receptor α is overexpressed in HeLa cells [23]. To our surprise, however, in pluripotent ES cells, PHBs are predominantly localized in mitochondria and do not actively shuttle in pluripotent mouse ES cells through a CRM1-dependent mechanism, although CRM1 is functional in pluripotent ES cells. Moreover, we have demonstrated that only mitochondrial PHB2 regulates ES cell differentiation since the mitochondria-targeted signal-mutated PHB2^AAAA^ localized in the cytoplasm and nucleus failed to inhibit ES cell differentiation. Our results unexpectedly revealed that PHB2 localized in the mitochondria but not in the nucleus and cytosol are crucial for the regulation of proliferation and differentiation of ES cells.

In addition to cell proliferation and differentiation, PHB2 also regulates mitochondrial morphogenesis and dynamics. Merkwirth *et al.* reported that OPA1, a dynamin-like GTPase that is a central component of the mitochondrial fusion and cristae remodeling machinery, is the major target of PHB2. PHB2-knockout MEFs show significant growth deficiency [8]. In this study, we have shown that knockdown of PHB2 induced loss of long isoforms of OPA1 and caused massive apoptosis in ES cells. Overexpression of PHB2 also induced loss of long isoforms of OPA1 and enlarged mitochondria in ES cells, although the effects on OPA1 were much milder than that of PHB2 knockdown. These observations suggested an imbalanced expression of PHBs caused aberrations of mitochondrial functions by modulating the processing of OPA1 protein. However, pluripotent ES cells are highly dependent on anaerobic glycolytic metabolism rather than the more efficient mitochondrial oxidative metabolism for energy production [28]. Thus, pluripotent ES cells overexpressing PHB2 may be capable of surviving even under these potential problems.

In differentiated cells, mitochondrial morphogenesis and dynamics are much more important than that in pluripotent ES cells. Mitra *et al.* reported that in normal kidney epithelial cells a hyperfused giant network of mitochondria is necessary for proper G_1_-S transition during the cell cycle [18]. Homozygous mutations in the genes required for mitochondrial dynamics also cause neuronal degeneration [29]. For example, mutations in the *OPA1* gene cause autosomal dominant optic atrophy [30], [31]. *MFN2*, one of the core genes in the mitochondrial fusion machinery, is responsible for a very common hereditary neuropathy, Charcot-Marie-Tooth disease [32]. These *in vivo* observations support our finding that PHB2, an upstream regulatory factor for OPA1, regulates neuronal differentiation of stem cells.

Although mature neurons are highly dependent on mitochondria because of their high-energy demands, neural stem/progenitor cells still have morphologically immature mitochondria, and most of them are fragmented similar to those in ES cells. During differentiation to mature neurons, mitochondria undergo morphological transition to fused hypertrophic state associated with enhancement of the mitochondrial transmembrane potential [33]. Therefore, pro-apoptotic changes in mitochondria caused by PHB2 overexpression could compromise survival of mature neurons, but not of neural stem/progenitor cells and pluripotent stem cells. In fact, most of the PHB2-overexpressing cells died after they differentiated into neurons, whereas undifferentiated cells survived (Figs. 4E–F, 4I–K, and 8F). Moreover, Feng *et al.* recently reported that an important regulatory transcription factor, Sox2, protected neural stem cells from apoptosis by upregulating the expression of survivin [34]. These observations suggest that neural stem cells, but not differentiated neurons, are protected by the Sox2-survivin pathway. Thus, overexpression of PHB2 may not induce apoptosis in neural stem cells.

In contrast to neuronal lineage differentiation, differentiation of ES cells to mesodermal lineages, such as cardiomyocytes, was not inhibited by exogenous PHB2. During the differentiation of ES cells into cardiomyocytes, the cellular metabolic system has to be transformed from anaerobic glycolytic metabolism, which is characteristic in ES and cancer cells, to the more efficient mitochondrial oxidative metabolism mandatory for the production of functional progenies of the cardiac lineage. Disruption of the electron transport chain by antimycin or rotenone interferes with the formation of mitochondrial organization, and the failure of energetic infrastructure results in defective sarcomerogenesis and contractile malfunction [35]. Maturation of mitochondria and their oxidative capacity was also evident during cardiomyogenic differentiation of cardiomyoblasts [36]. Interestingly, Hom *et al.* reported that the mitochondrial permeability transition pore (mPTP) was open in early embryonic heart; however, apoptotic factors, including cytochrome *c* and apoptosis-inducing factor, were not released from the mitochondria to the cytosol in E9.5 myocytes [37], which indicates that mPTP opening in early embryonic heart is nonpathologic. Their results suggest that differentiating cardiomyocytes from the progenitor cells could be more resistant to the pathologic effects observed during differentiation.

In this study, we found that PHB2 highly expressed in mitochondria is essential for the survival and proliferation of pluripotent stem cells. The expression level of PHB2 also modulates the differentiation of ES cells. Our results revealed that *PHB2*, an essential gene for mitochondrial homeostasis, functions as a crucial molecule in mitochondria that regulates proliferation and lineage specification during stem cell differentiation.

## Materials and Methods

### Cell Culture

The mouse ES cell line D3 was purchased from the American Tissue Culture Collection (ATCC). EBRTcH3, a mouse ES cell line for knock-in experiments [14], was a kind gift from Drs. Hitoshi Niwa and Shinji Masui (Riken, Japan). These cells were maintained on mitomycin C-treated mouse embryonic fibroblast (MEF) feeder layer in ES medium (DMEM with high glucose [Wako]), 2 mM l-glutamine, 0.1 mM nonessential amino acids (GIBCO), 0.1 mM 2-mercaptoethanol, 15% FBS (Nichirei Biosciences), 1,000 IU/ml ESGRO (Chemicon), and penicillin/streptomycin (Sigma). MEFs were prepared as described previously [38], and maintained in MEF medium (DMEM with low glucose [WAKO], 2 mM l-glutamine, 12.5% FBS, and penicillin/streptomycin). A human iPS cell line (201B7) was obtained from Riken Cell Bank and cultured as described [40]. The experimental protocols for the preparation of MEFs from ICR mouse embryos and use of human iPS cells were approved by the Animal Experiment Committee and Ethical committee of National Institute of Advanced Industrial Science and Technology (AIST), respectively. For retrovirus propagation, Plat-E cells [39] were maintained in DMEM with low glucose, 10% FBS, 10 µg/ml blasticidin (Invitrogen), 1 µg/ml puromycin (Sigma), and penicillin/streptomycin (Sigma).

### Plasmid Construction

For the construction of an expression vector for PHB2, the full-length coding region was amplified by polymerase chain reaction (PCR) with pCMV-SPORT-PHB2-Flag vector [23] as the template. The following primers were used for PCR amplification: human PHB2-5′: CTCCTCGAGGCCACCATGGCCCAGAACTTGAAG; human PHB2-3′: GAGGCGGCCGCTTAGTGATGGTGATGGTG. For cDNA amplification, a high-fidelity PCR enzyme, KOD-Plus (TOYOBO), was used. The resulting fragments were digested by *Xho*I and *Not*I, and subcloned into the *Xho*I and *Not*I sites of the pPthC vector [14].

Tc-regulated PHB-expressing ES cell lines were established according to the method of Masui *et al.* [14]. An EB3 cell–derived mouse ES cell line, EBRTcH3, which has a Tet-off cassette in the ROSA26 locus [14], was cotransfected with a targeting vector, pPthC-PHB2-Flag, and a Cre recombinase expressing vector, pCAGGS-Cre, by using Lipofectamine 2000 (Invitrogen) and cultured in the presence of 1.5 µg/ml puromycin (Sigma) and 1 µg/ml Tc. The established ES clones were cultured in ES medium with or without Tc, and the expression of the exogenous gene was examined by immunoblotting (Fig. 3A, 3B). The expression of the exogenous gene was also confirmed by coexpression of the fluorescent protein, Venus. The Tc-regulated PHB2-expressing ES cell lines were maintained in ES medium on a feeder cell layer of puromycin-resistant, mitomycin C–inactivated MEFs (DR4 line from ATCC). The medium was changed daily, and the cells were passaged every 3 days.

To obtain human iPS clones stably expressing PHB2, pCMV-SPORT-PHB2-Flag vector was digested with *Hind*III, blunted with T4 polymerase, digested with *Eco*RI, and subcloned into the *Eco*RI and *Eco*RV sites of the pCAG-IP vector [41]. Human iPS cells (201B7) were transfected with pCAG-IP-PHB2-Flag vector using Lipofectamine 2000 (Invitrogen). Human iPS cell clones stably expressing PHB2-Flag were selected in the presence of 0.4 µg/ml of puromycin on DR4 MEFs (ATCC). The expression of PHB2-Flag was confirmed by immunoblotting (Fig. S1A).

For knockdown experiments, the retroviral vectors pSinSi-DK-PHB2 shRNA and the negative control vector were constructed by inserting the following sense-loop-antisense DNA sequences into *Bam*HI and *Cla*I sites of the pSinSi-DK-I vector (Takara): PHB2 shRNA, sense: 5′- CTAGAGAACCGAATCTATCTCAACACAGGGAAGCGAGTCTGTGTGAGATAGATTCGGTCTTTTTCCTGCA-3′, antisense: 5′-GGAAAAAAGAACCGAATCTACTTATCTCACACAGACTCGCTTCCCTGTGTGTGAGATAGATTCGGTTCT-3′; and negative control-1 shRNA, sense: 5′-CTAGAGTCTTAATCGCGTATAAGGCCACAGGGAAGCGAGTCTGGCCTTATACGCGATTAAGACTTTTTTCCTGCA-3′, antisense: 5′-GGAAAAAAGTCTTAATCGCGTATAAGGCCAGACTCGCTTCCCTGTGGCCTTATACGCGATTAAGACT-3′. Knockdown of PHB2 was confirmed by transient transfection of pSinSi-DK-PHB2 shRNA vector followed by immunofluorescence staining (Fig. 2A).

A Tet-off-based PHB2 miR expression vector was constructed with the same target region of PHB2 as pSinSi-DK-PHB2 shRNA vector. The following sense-loop-antisense DNA sequences were annealed and inserted into *Sal*I and *Xho*I sites of the pcdNA6.2/GW-EGFPmiR vector: PHB2 shRNA, sense: 5′- TGCTGCTGTGAGATAGATTCGGTTCTGTTTTGGCCACTGACTGACAGAACCGACTATCTCACAG-3′, antisense: 5′- CCTGCTGTGAGATAGTCGGTTCTGTCAGTCAGTGGCCAAAACAGAACCGAATCTATCTCACAGC-3′. As for the control vector, pcDNA6.2-GW/miR-neg (Invitrogen) was used as negative control. The PHB2 and the negative control miR regions were further amplified with the following primers: sense: 5′- AAACTCGAGTAGGCGTGTACGGTGGGAGGCCTATATAAGCAGAGCTCGTTTAGTGAACCGTCAGATCGCCTGGAGAATTCGCCACCCTGGAGGCTTGCTGAAG-3′, antisense: 5′- TTTGCGGCCGCACACACAAAAAACCAACACACAGATGTAATGAAAATAAAGATATTTTATTGGGCCATTTGTTCCATGTGA-3′.

The amplified fragments were digested with *Not*I and *Xho*I, and ligated into the *Not*I and *Xho*I sites of pPthC vector to generate pPthC-PHB2 miR and the control miR vector. Knockdown of PHB2 was monitored by immunoblotting (Fig. S1B).

For the construction of shRNA-insensitive mouse PHB2-GFP expression vector, the GFP expression vectors, PHB2wt-eGFP and PHB2AAAA-eGFP (kind gift from Dr. Langer) [8], were mutated by inverse PCR using KOD plus Neo (TOYOBO) with the following primers: 5′-TACCTGACTGCTGACAACCTTGTGCTGAA-3′, antisense: 5′- TATACGATTCTGTGATGTGGCGATCG-3′. The resulting fragments were digested by *Eco*RI and *Not*I, and subcloned into the *Eco*RI and *Not*I sites of the pCAG-IP vector (provided by Dr. Koide) [41].

A Tet-off-based PHB2 miRNA expression vector was constructed using the same target sequence of PHB2 as pSinSi-DK-I-PHB2 shRNA vector. The following sense-loop-antisense DNA sequences were annealed and inserted into the *Sal*I and *Xho*I sites of the pcDNA6.2/GW-EGFP miR vector, sense: 5′-TGCTGCTGTGAGATAGATTCGGTTCTGTTTTGGCCACTGACTGACAGAACCGACTATCTCACAG-3′, antisense: 5′-CCTGCTGTGAGATAGTCGGTTCTGTCAGTCAGTGGCCAAAACAGAACCGAATCTATCTCACAGC-3′. The PHB2 miR region was further amplified by PCR using KOD FX Neo (TOYOBO) with the following primers, sense: 5′- AAACTCGAGTAGGCGTGTACGGTGGGAGGCCTATATAAGCAGAGCTCGTTTAGTGAACCGTCAGATCGCCTGGAGAATTCGCCACCCTGGAGGCTTGCTGAAG -3′, antisense: 5′- TTTGCGGCCGCACACACAAAAAACCAACACACAGATGTAATGAAAATAAAGATATTTTATTGGGCCATTTGTTCCATGTGA-3′. The PCR amplicon was digested with *Not*I and *Xho*I, and ligated into the *Not*I and *Xho*I sites of pPthC vector [14] to generate pPthC-PHB2 miR vector. Tc-regulated PHB miR-expressing ES cell lines were established as described above.

### Differentiation of ES Cells

For the induction of cardiomyocytes, the mouse ES cell line EBRTcH3, carrying the PHB2 transgene under the control of a Tc-regulated promoter, was dissociated with trypsin and aggregated at 1,000 cells per well on a 96-well low-cell-binding microplate (Nunc) in ES medium without LIF for 2 days culture. The resulting embryoid bodies (EBs) were further cultured in suspension for 2 more days. Then, EBs were cultured on a gelatin-coated dish for 12 days in ES medium without LIF. Neuronal cells were differentiated according to a previous report [15] using the above-mentioned EBRTcH3 cell line with minor modification. The EBs were cultured in suspension for 5 days followed by an adhesion culture for 4 days. For definitive endoderm differentiation, the ES cells were aggregated at 1,000 cells per well in ES medium without LIF. After 3 days culture, the EBs were plated on a gelatin-coated dish with ES medium supplemented with 100 ng/ml activin A (R&D Systems) and incubated for 24 h. On the next day, the medium was changed to ES medium without LIF and cultured for 2 more days. After differentiation, cells were immunostained with lineage-specific marker antibodies, and the immunopositive cells were counted to calculate the differentiation efficiency. For definitive endoderm differentiation of human iPS cells, a human iPS cell line, 201B7, was differentiated into endoderm cells according to a previously published method [42].

### Alkaline Phosphatase Staining

Mouse ES cells cultured on gelatin-coated dishes were washed twice with PBS and fixed in 3.7% formaldehyde/PBS for 5 min at room temperature. The cells were washed twice with PBS and incubated with BM purple AP substrate (Roche) for 30 min at room temperature.

### Subcellular Fractionation

Mitochondria from mouse ES cells were fractionated with Mitochondria Purification Kit (Thermo Scientific) according to the manufacturer's protocol with some modifications. Briefly, mouse ES cells (D3) cultured on gelatin-coated dish without feeder cells were washed twice with PBS, dissociated with trypsin/EDTA, and harvested by centrifugation at 1,000 rpm for 5 min at room temperature. ES cells (2×10^7^ cells) were washed with PBS and suspended in solution A supplemented with protease inhibitor cocktail (Complete, Roche). The cells were incubated on ice for 2 min, homogenized with a Dounce homogenizer, and centrifuged at 700×*g* for 5 min at 4°C. The precipitated fraction was washed with solution A, and used as the nuclear fraction. The supernatant was centrifuged at 700×*g* for 5 min to further remove nucleus and debris, and centrifuged again at 12,000×*g* for 15 min 4°C. The supernatant fraction was used as the cytosolic fraction. The precipitated fraction was washed with solution A and used as the mitochondrial fraction. The nuclear and mitochondrial precipitated samples were resuspended in solubilization solution (20 mM Tris-HCl [pH 7.4], 150 mM NaCl, 1 mM EDTA, 1% NP40, and Complete) and rotated at 4°C overnight. The solubilized proteins were recovered by centrifugation at 12,000×*g* for 10 min at 4°C.

### Immunoblotting and Immunofluorescence Staining

For the preparation of cell extracts, mouse ES cells were lysed in a lysis buffer (20 mM Tris-HCl [pH 7.4], 150 mM NaCl, 1 mM EDTA, 1% Nonidet P-40, 1 mM Na_3_VO_4_, 25 mM NaF, and 25 mM β-glycerophosphate) supplemented with a protein inhibitor cocktail (Complete, Roche), and rotated at 4°C for 30 min. After centrifugation at 13,200 rpm for 10 min at 4°C, the supernatant was collected and protein concentration was determined with a Protein Assay Kit (Bio-Rad). Thirty micrograms of the sample was boiled in SDS sample buffer, resolved using SDS-PAGE, and transferred to polyvinylidene difluoride membranes. The membranes were blocked with 5% skim milk in TBS-T and incubated with first antibodies. After incubation with horseradish-conjugated secondary antibody, the blots were incubated with an enhanced chemiluminescent assay reagent (SuperSignal West Femto, Pierce) for 5 min at room temperature, and the protein bands were visualized using an LAS 1000 Pro Image Analyzer (Fuji Film). For quantitative analysis, the protein bands were further analyzed using Image Gauge software (Fuji Film).

Immunofluorescence staining was performed as described previously [43]. Confocal images of ES and differentiated cells were obtained using a FV1000 laser scanning confocal microscope (Olympus) equipped with a 40× UPlanSApo 0.95 objective lens. To analyze the subcellular localization of PHBs, a 60× UPlanSApo 1.35 or a 100× UPlanSApo 1.4 oil immersion objective lens was used with the same confocal system. For the detection of apoptosis of cells after shRNA-based knockdown of PHB2, DNA fragmentation was detected by the TUNEL method (DeadEnd™ Fluorometric TUNEL System, Promega) was used according to the manufacturer's instruction.

The antibodies used in this study were follows: PHB1 (BioVision, 3805-100), PHB2 (Bethyl Laboratories, A300-658), Oct4 (Santa Cruz, sc-9081; Abcam, ab19857), Nanog (ReproCELL, RCAB0001P), GFP (Santa Cruz, sc-8334), Flag M2 (Sigma, F3165), TuJ1 (Covance, MMS-435P), Neurod1 (Abcam, ab60704), MAP2 (Abcam, AB32454), Nestin (Millipore, MAB353), cardiac troponin T (cTnT; NeoMarkers, MS-295-P1), Foxa2 (Santa Cruz, sc-6554), Sox17 (Neuromics, GT15094), Smad4 (Santa Cruz, sc-7966), and α-tubulin (Sigma, T9026). To detect mitochondria, MitoTracker (Molecular Probes) was used according to the manufacturer's instruction. The secondary antibodies used for the immunofluorescence study were anti-mouse and anti-rabbit IgG-conjugated Alexa 488 or Alexa 594 (Molecular Probes). All immunofluorescence images other than the confocal images were obtained using an Olympus IX70 microscope equipped with CoolSNAP HQ^2^ (Photometrics) and processed using MetaMorph software (Molecular Devices).

### ATP Biogenesis and ROS Production

ATP biosynthesis was measured using an ATP Bioluminescence Assay Kit HS II (Roche) following the manufacturer's instruction. For the analysis of ROS production, ES cells were incubated in a medium containing 2 µM dihydroethidium (Molecular Probes) at 37°C in dark for 15 min, washed with PBS, and suspended in PBS containing 0.5% BSA. The fluorescence intensity of 30,000 cells was recorded with a BD Biosciences LSR II (BD Biosciences, MD).

### Membrane Potential

The membrane potential of mitochondria in ES cells was measured with a confocal microscope. The cells were incorporated with a cytofluorimetric, lipophilic cationic dye, JC-1, as described [44].

### Electron Microscopy

ES cells were fixed with 0.1 M sodium cacodylate buffer (pH 7.4) containing 3% paraformaldehyde and 2.5% glutaraldehyde, and washed with 0.2 M sodium cacodylate buffer 3 times. The cells were postfixed with 1% osmium tetroxide for 30 min, dehydrated through an ethanol and acetone series, and then embedded in epoxy resin. Ultrathin sections cut at 80–90 nm thickness were stained with uranyl acetate and lead citrate, and observed under a transmission electron microscope (JEM-200CX; JEOL, Tokyo, http://www.jeol.com).

### Statistical Analysis

Statistical significance was determined by Student's *t*-test. Differences were considered significant at P<0.05. All statistical analysis was performed using the data analysis package included in Microsoft Excel 2007 software.

## Supporting Information

Figure S1 **Expression of prohibitin 2 (PHB2) in PHB2-overexpressing embryonic stem (ES) cells and PHB2-knockdown ES cells.** (A) Expression level of PHB2 in human induced pluripotent stem (iPS) cells stably expressing C-terminally Flag-tagged PHB2. (B) Expression level of endogenous PHB2 in mouse ES cells expressing tetracycline (Tc)-regulated PHB2 shRNA.
(TIF)

Movie S1 **Time laps analysis of mitochondria in ES cells.** EBRTcH3 cells carrying the *PHB2* gene under the control of a Tc-regulated promoter were cultured with Tc (S2) or without Tc (S1) for 4 days, and subjected to time laps analysis using a confocal microscope. Similarly, the EBRTcH3 cells carrying the *PHB2*-specific *miR* under the control of a Tc-regulated promoter were cultured with (S4) or without (S3) Tc for 4 days, and subjected to time laps analysis using a confocal microscope. *PHB2* gene and *PHB2 miR* are expressed in the absence of Tc. Scale bar, 5 µm.
(AVI)

Movie S2 **Time laps analysis of mitochondria in ES cells.** EBRTcH3 cells carrying the *PHB2* gene under the control of a Tc-regulated promoter were cultured with Tc (S2) or without Tc (S1) for 4 days, and subjected to time laps analysis using a confocal microscope. Similarly, the EBRTcH3 cells carrying the *PHB2*-specific *miR* under the control of a Tc-regulated promoter were cultured with (S4) or without (S3) Tc for 4 days, and subjected to time laps analysis using a confocal microscope. *PHB2* gene and *PHB2 miR* are expressed in the absence of Tc. Scale bar, 5 µm.
(AVI)

Movie S3 **Time laps analysis of mitochondria in ES cells.** EBRTcH3 cells carrying the *PHB2* gene under the control of a Tc-regulated promoter were cultured with Tc (S2) or without Tc (S1) for 4 days, and subjected to time laps analysis using a confocal microscope. Similarly, the EBRTcH3 cells carrying the *PHB2*-specific *miR* under the control of a Tc-regulated promoter were cultured with (S4) or without (S3) Tc for 4 days, and subjected to time laps analysis using a confocal microscope. *PHB2* gene and *PHB2 miR* are expressed in the absence of Tc. Scale bar, 5 µm.
(AVI)

Movie S4 **Time laps analysis of mitochondria in ES cells.** EBRTcH3 cells carrying the *PHB2* gene under the control of a Tc-regulated promoter were cultured with Tc (S2) or without Tc (S1) for 4 days, and subjected to time laps analysis using a confocal microscope. Similarly, the EBRTcH3 cells carrying the *PHB2*-specific *miR* under the control of a Tc-regulated promoter were cultured with (S4) or without (S3) Tc for 4 days, and subjected to time laps analysis using a confocal microscope. *PHB2* gene and *PHB2 miR* are expressed in the absence of Tc. Scale bar, 5 µm.
(AVI)

## References

[1] Ng HH, Surani MA (2011) The transcriptional and signalling networks of pluripotency. Nat Cell Biol 13: 490–496. 21540844 10.1038/ncb0511-490

[2] Niwa H, Ogawa K, Shimosato D, Adachi K (2009) A parallel circuit of LIF signalling pathways maintains pluripotency of mouse ES cells. Nature 460: 118–122. 19571885 10.1038/nature08113

[3] Rehman J (2010) Empowering self-renewal and differentiation: the role of mitochondria in stem cells. J Mol Med (Berl) 88: 981–986. 20809088 10.1007/s00109-010-0678-2 PMC3006229

[4] Facucho-Oliveira JM, St John JC (2009) The relationship between pluripotency and mitochondrial DNA proliferation during early embryo development and embryonic stem cell differentiation. Stem Cell Rev 5: 140–158. 19521804 10.1007/s12015-009-9058-0

[5] Intoh A, Kurisaki A, Fukuda H, Asashima M (2009) Separation with zwitterionic hydrophilic interaction liquid chromatography improves protein identification by matrix-assisted laser desorption/ionization-based proteomic analysis. Biomed Chromatogr 23: 607–614. 19280682 10.1002/bmc.1159

[6] Artal-Sanz M, Tavernarakis N (2009) Prohibitin and mitochondrial biology. Trends Endocrinol Metab 20: 394–401. 19733482 10.1016/j.tem.2009.04.004

[7] Merkwirth C, Langer T (2009) Prohibitin function within mitochondria: essential roles for cell proliferation and cristae morphogenesis. Biochim Biophys Acta 1793: 27–32. 18558096 10.1016/j.bbamcr.2008.05.013

[8] Merkwirth C, Dargazanli S, Tatsuta T, Geimer S, Lower B, et al (2008) Prohibitins control cell proliferation and apoptosis by regulating OPA1-dependent cristae morphogenesis in mitochondria. Genes Dev 22: 476–488. 18281461 10.1101/gad.460708 PMC2238669

[9] Thuaud F, Ribeiro N, Nebigil CG, Desaubry L (2013) Prohibitin ligands in cell death and survival: mode of action and therapeutic potential. Chem Biol 20: 316–331. 23521790 10.1016/j.chembiol.2013.02.006 PMC7111013

[10] Czarnecka AM, Campanella C, Zummo G, Cappello F (2006) Mitochondrial chaperones in cancer: from molecular biology to clinical diagnostics. Cancer Biol Ther 5: 714–720. 16861898 10.4161/cbt.5.7.2975

[11] Park SE, Xu J, Frolova A, Liao L, O'Malley BW, et al (2005) Genetic deletion of the repressor of estrogen receptor activity (REA) enhances the response to estrogen in target tissues in vivo. Mol Cell Biol 25: 1989–1999. 15713652 10.1128/MCB.25.5.1989-1999.2005 PMC549370

[12] Akama K, Horikoshi T, Nakayama T, Otsu M, Imaizumi N, et al (2011) Proteomic identification of differentially expressed genes in neural stem cells and neurons differentiated from embryonic stem cells of cynomolgus monkey (Macaca fascicularis) in vitro. Biochim Biophys Acta 1814: 265–276. 21047566 10.1016/j.bbapap.2010.10.009

[13] Baharvand H, Hajheidari M, Ashtiani SK, Salekdeh GH (2006) Proteomic signature of human embryonic stem cells. Proteomics 6: 3544–3549. 16758447 10.1002/pmic.200500844

[14] Masui S, Shimosato D, Toyooka Y, Yagi R, Takahashi K, et al (2005) An efficient system to establish multiple embryonic stem cell lines carrying an inducible expression unit. Nucleic Acids Res 33: e43. 15741176 10.1093/nar/gni043 PMC552969

[15] Watanabe K, Kamiya D, Nishiyama A, Katayama T, Nozaki S, et al (2005) Directed differentiation of telencephalic precursors from embryonic stem cells. Nat Neurosci 8: 288–296. 15696161 10.1038/nn1402

[16] Rastogi S, Joshi B, Fusaro G, Chellappan S (2006) Camptothecin induces nuclear export of prohibitin preferentially in transformed cells through a CRM-1-dependent mechanism. J Biol Chem 281: 2951–2959. 16319068 10.1074/jbc.M508669200

[17] Watanabe M, Masuyama N, Fukuda M, Nishida E (2000) Regulation of intracellular dynamics of Smad4 by its leucine-rich nuclear export signal. EMBO Rep 1: 176–182. 11265759 10.1093/embo-reports/kvd029 PMC1084261

[18] Mitra K, Wunder C, Roysam B, Lin G, Lippincott-Schwartz J (2009) A hyperfused mitochondrial state achieved at G1-S regulates cyclin E buildup and entry into S phase. Proc Natl Acad Sci U S A 106: 11960–11965. 19617534 10.1073/pnas.0904875106 PMC2710990

[19] Olichon A, Baricault L, Gas N, Guillou E, Valette A, et al (2003) Loss of OPA1 perturbates the mitochondrial inner membrane structure and integrity, leading to cytochrome c release and apoptosis. J Biol Chem 278: 7743–7746. 12509422 10.1074/jbc.C200677200

[20] Griparic L, van der Wel NN, Orozco IJ, Peters PJ, van der Bliek AM (2004) Loss of the intermembrane space protein Mgm1/OPA1 induces swelling and localized constrictions along the lengths of mitochondria. J Biol Chem 279: 18792–18798. 14970223 10.1074/jbc.M400920200

[21] Frezza C, Cipolat S, Martins de Brito O, Micaroni M, Beznoussenko GV, et al (2006) OPA1 controls apoptotic cristae remodeling independently from mitochondrial fusion. Cell 126: 177–189. 16839885 10.1016/j.cell.2006.06.025

[22] Montano MM, Ekena K, Delage-Mourroux R, Chang W, Martini P, et al (1999) An estrogen receptor-selective coregulator that potentiates the effectiveness of antiestrogens and represses the activity of estrogens. Proc Natl Acad Sci U S A 96: 6947–6952. 10359819 10.1073/pnas.96.12.6947 PMC22022

[23] Kasashima K, Ohta E, Kagawa Y, Endo H (2006) Mitochondrial functions and estrogen receptor-dependent nuclear translocation of pleiotropic human prohibitin 2. J Biol Chem 281: 36401–36410. 17008324 10.1074/jbc.M605260200

[24] Sun L, Cao X, Liu B, Huang H, Wang X, et al (2011) CaMK IV phosphorylates prohibitin 2 and regulates prohibitin 2-mediated repression of MEF2 transcription. Cell Signal 23: 1686–1690. 21689744 10.1016/j.cellsig.2011.06.005 PMC7127762

[25] Heron-Milhavet L, Mamaeva D, Rochat A, Lamb NJ, Fernandez A (2008) Akt2 is implicated in skeletal muscle differentiation and specifically binds Prohibitin2/REA. J Cell Physiol 214: 158–165. 17565718 10.1002/jcp.21177

[26] Wang S, Fusaro G, Padmanabhan J, Chellappan SP (2002) Prohibitin co-localizes with Rb in the nucleus and recruits N-CoR and HDAC1 for transcriptional repression. Oncogene 21: 8388–8396. 12466959 10.1038/sj.onc.1205944

[27] Wang S, Nath N, Fusaro G, Chellappan S (1999) Rb and prohibitin target distinct regions of E2F1 for repression and respond to different upstream signals. Mol Cell Biol 19: 7447–7460. 10523633 10.1128/mcb.19.11.7447 PMC84738

[28] Chen CT, Hsu SH, Wei YH (2012) Mitochondrial bioenergetic function and metabolic plasticity in stem cell differentiation and cellular reprogramming. Biochim Biophys Acta 1820: 571–576. 21983491 10.1016/j.bbagen.2011.09.013

[29] Detmer SA, Chan DC (2007) Functions and dysfunctions of mitochondrial dynamics. Nat Rev Mol Cell Biol 8: 870–879. 17928812 10.1038/nrm2275

[30] Alexander C, Votruba M, Pesch UE, Thiselton DL, Mayer S, et al (2000) OPA1, encoding a dynamin-related GTPase, is mutated in autosomal dominant optic atrophy linked to chromosome 3q28. Nat Genet 26: 211–215. 11017080 10.1038/79944

[31] Delettre C, Lenaers G, Griffoin JM, Gigarel N, Lorenzo C, et al (2000) Nuclear gene OPA1, encoding a mitochondrial dynamin-related protein, is mutated in dominant optic atrophy. Nat Genet 26: 207–210. 11017079 10.1038/79936

[32] Zuchner S, Mersiyanova IV, Muglia M, Bissar-Tadmouri N, Rochelle J, et al (2004) Mutations in the mitochondrial GTPase mitofusin 2 cause Charcot-Marie-Tooth neuropathy type 2A. Nat Genet 36: 449–451. 15064763 10.1038/ng1341

[33] Voccoli V, Colombaioni L (2009) Mitochondrial remodeling in differentiating neuroblasts. Brain Res 1252: 15–29. 19071097 10.1016/j.brainres.2008.11.026

[34] Feng R, Zhou S, Liu Y, Song D, Luan Z, et al (2013) Sox2 protects neural stem cells from apoptosis via up-regulating survivin expression. Biochem J 450: 459–468. 23301561 10.1042/BJ20120924

[35] Chung S, Dzeja PP, Faustino RS, Perez-Terzic C, Behfar A, et al (2007) Mitochondrial oxidative metabolism is required for the cardiac differentiation of stem cells. Nat Clin Pract Cardiovasc Med 4 Suppl 1: S60–67. 17230217 10.1038/ncpcardio0766 PMC3232050

[36] Comelli M, Domenis R, Bisetto E, Contin M, Marchini M, et al (2011) Cardiac differentiation promotes mitochondria development and ameliorates oxidative capacity in H9c2 cardiomyoblasts. Mitochondrion 11: 315–326. 21147273 10.1016/j.mito.2010.12.007

[37] Hom JR, Quintanilla RA, Hoffman DL, de Mesy Bentley KL, Molkentin JD, et al (2011) The permeability transition pore controls cardiac mitochondrial maturation and myocyte differentiation. Dev Cell 21: 469–478. 21920313 10.1016/j.devcel.2011.08.008 PMC3175092

[38] Hogan B, Beddington R, Constantini F, Lacy E (1994) Manipulating the Mouse Embryo: A Laboratory Manual. NY: Cold Spring Harbor Laboratory Press.

[39] Morita S, Kojima T, Kitamura T (2000) Plat-E: an efficient and stable system for transient packaging of retroviruses. Gene Ther 7: 1063–1066. 10871756 10.1038/sj.gt.3301206

[40] Takahashi K, Tanabe K, Ohnuki M, Narita M, Ichisaka T, et al (2007) Induction of pluripotent stem cells from adult human fibroblasts by defined factors. Cell 131: 861–872. 18035408 10.1016/j.cell.2007.11.019

[41] Yoshida-Koide U, Matsuda T, Saikawa K, Nakanuma Y, Yokota T, et al (2004) Involvement of Ras in extraembryonic endoderm differentiation of embryonic stem cells. Biochem Biophys Res Commun 313: 475–481. 14697213 10.1016/j.bbrc.2003.11.138

[42] Kunisada Y, Tsubooka-Yamazoe N, Shoji M, Hosoya M (2012) Small molecules induce efficient differentiation into insulin-producing cells from human induced pluripotent stem cells. Stem Cell Res 8: 274–284. 22056147 10.1016/j.scr.2011.10.002

[43] Kurisaki A, Kurisaki K, Kowanetz M, Sugino H, Yoneda Y, et al (2006) The mechanism of nuclear export of Smad3 involves exportin 4 and Ran. Mol Cell Biol 26: 1318–1332. 16449645 10.1128/MCB.26.4.1318-1332.2006 PMC1367208

[44] Mandal S, Lindgren AG, Srivastava AS, Clark AT, Banerjee U (2011) Mitochondrial function controls proliferation and early differentiation potential of embryonic stem cells. Stem Cells 29: 486–495. 21425411 10.1002/stem.590 PMC4374603

