# Supplementary figures and images for: Prohibitin 2 Regulates the Proliferation and Lineage-Specific Differentiation of Mouse Embryonic Stem Cells in Mitochondria
Source: PLoS One. 2014 Apr 7;9(4):e81552. doi: 10.1371/journal.pone.0081552 (PMC3977857; doi:10.1371/journal.pone.0081552)

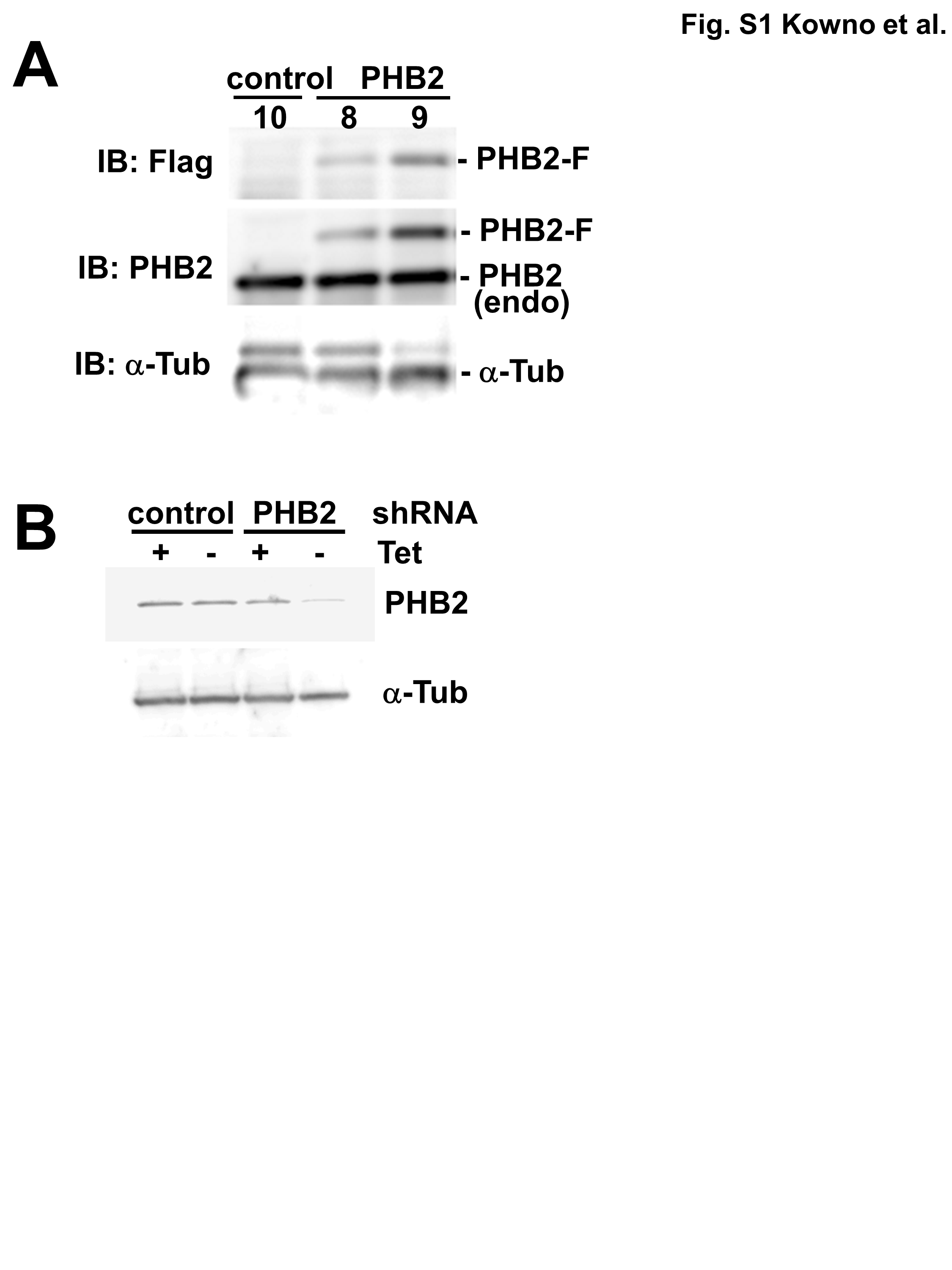

Supplement: Figure S1 — Expression of prohibitin 2 (PHB2) in PHB2-overexpressing embryonic stem (ES) cells and PHB2-knockdown ES cells. (A) Expression level of PHB2 in human induced pluripotent stem (iPS) cells stably expressing C-terminally Flag-tagged PHB2. (B) Expression level of endogenous PHB2 in mouse ES cells expressing tetracycline (Tc)-regulated PHB2 shRNA. (TIF) [file pone.0081552.s001.tif]
